# Diagnostic Value of SALL4 and OCT3/4 in Pediatric Testicular Tumors

**DOI:** 10.3390/diagnostics14131454

**Published:** 2024-07-08

**Authors:** Ovidiu Bîcă, Carmen Iulia Ciongradi, Marius Ivănuță, Victor Ianole, Ioan Sârbu, Elena Cojocaru, Delia Elena Bîcă, Ludmila Lozneanu

**Affiliations:** 1 2nd Department of Surgery—Pediatric Surgery and Orthopedics, “Grigore T. Popa” University of Medicine and Pharmacy, 700115 Iași, Romania; 2 Department of Urology, “Grigore T. Popa” University of Medicine and Pharmacy, 700115 Iași, Romania; marius-f-ivanuta@d.umfiasi.ro; 3 Department of Morphofunctional Sciences I—Pathology, “Grigore T. Popa” University of Medicine and Pharmacy, 700115 Iaşi, Romania; 4 Department of Clinical Pharmacology, “Grigore T. Popa” University of Medicine and Pharmacy, 700115 Iași, Romania; 5 Department of Morpho-Functional Sciences I—Histology, “Grigore T. Popa” University of Medicine and Pharmacy, 700115 Iași, Romania

**Keywords:** pediatric, testicular, tumors, immunohistochemistry, SALL4, OCT3/4

## Abstract

Testicular tumors (TTs) are rare in children, posing diagnostic and therapeutic challenges. This retrospective study evaluates the diagnostic and prognostic utility of SALL4 and OCT3/4 in pediatric TTs. We analyzed 18 cases of different types of TTs using immunohistochemistry (IHC) to assess SALL4 (Spalt-like transcription factor 4) and OCT3/4 (Octamer binding transcription factor 3/4) expression. SALL4 was positive in 83.3% of tumors, while OCT3/4 was positive in 38.9% of tumors, with a significantly higher prevalence in patients aged 12–18 years compared to those aged 0–11 years (***p* = 0.013**). Mixed germinal cell tumors were significantly more frequently associated with OCT3/4 (***p* = 0.003**), and a high immunostaining expression for SALL4 was observed primarily in yolk sac tumors and embryonal carcinoma. Our findings suggest that SALL4 and OCT3/4 immunostaining can aid in accurate diagnosis and treatment planning, and underscores the importance of OCT3/4 as a predictive factor in pediatric testicular tumors, highlighting its substantial correlation with tumor type and its impact on treatment response. These markers may guide personalized therapeutic strategies, potentially improving patient outcomes.

## 1. Introduction

Testicular tumors (TTs) are uncommon across the pediatric population, representing around 1% to 2% of all solid tumors, with an annual incidence of between 1.6 and 2 per 100.000 children [1]. The new World Health Organization (WHO) 2022 [2] classification system divides germ cell testicular tumors (GCTTs) into two main groups: GCTs derived from germ cell neoplasia in situ (GCNIS-derived) and lesions that develop unrelated to GCNIS (non-GCNIS-derived) [2]. Regarding bimodal disease distribution, the first peak occurs between the ages of 0 and 11 years and is mostly made up of non-GCNIS-derived tumors, such as teratoma-prepubertal-type and yolk sac tumors (YSTs). The second peak occurs during puberty (after 12 years of age) and is mostly made up of GCNIS-derived tumors, such as seminomas, teratoma-postpubertal type, and mixed germ cell tumors (mixed GCT) [3]. The most common types of TTs in children are prepubertal teratomas (50%), followed by YSTs (15%). Other tumors include epidermoid cysts (15%) and stromal tumors (Leydig cell and Sertoli cell), which amount to almost 10%. Few significant studies have highlighted the importance of immunohistochemical (IHC) markers in pediatric cases, despite GCT accounting for 3% of childhood cancers [4]. 

SALL4 (Spalt like transcription factor 4) is identified as a member of the mammalian homologs of the spalt (sal) homeotic gene from Drosophila and forms a regulatory network with other pluripotency-associated transcription factors, such as OCT3/4 and NANOG, which are essential for genes control and processes involved in the self-renewal of embryonic stem cells [5]. The application of IHC methods to determine SALL4 expression has proven useful in diagnosing primary and metastatic testicular GCT. A SALL4-positive expression was seen in almost all non-trophoblastic GCT (seminoma, embryonal carcinoma (EC) and YST) and in germ cell neoplasia in situ, and in nearly 80% of choriocarcinomas and 60% of immature teratoma [6].

OCT3/4 (Octamer transcription factor 3/4) is a transcription factor encoded by the POUF5 gene (POU domain, class 5) located on chromosome 6p21 and is down-regulated during cellular differentiation into endoderm and mesoderm [7]. OCT3/4 can act also as an oncogene in various types of cancer. OCT3/4 regulates tumor-initiating cells’ functions, such as self-renewal, survival, epithelial–mesenchymal transition, metastasis, and the development of treatment resistance [8,9]. OCT3/4 is expressed in malignant germ cells with pluripotent potential and is a useful marker for tumors derived from primary and secondary GCTs, with nearly 100% expression in seminomas, EC and germ cell components of gonadoblastoma [10].

The main goal of this study was to examine the expression patterns of specific IHC markers (SALL4 and OCT3/4) in different types of TTs and to analyze the association between the expression of these markers and clinicopathological features that can influence treatment and prognosis.

## 2. Materials and Methods

### 2.1. Patients

This retrospective study analyzed pediatric patients under 18 years of age with testicular and paratesticular tumors diagnosed and treated at our hospital from January 2003 to January 2023. Patient selection was conducted using the ICD-10 coding system, and medical chart records were reviewed to collect epidemiological and clinical data, pre-operative serum tumor markers levels, surgical procedures (conservative or radical orhiectomy), medical oncology therapy (chemotherapy protocols and number of chemotherapy sessions), clinical outcomes (evolution and follow up period), and pathological characteristics of patients (tumor size and histopathological type). The exclusion criteria were as follows: age over 18 years; patients who presented for second opinion evaluation of paraffin blocks; patients requesting paraffin blocks from the pathology department to other hospitals; and patients diagnosed with secondary testicular tumors (1 case of Burkitt lymphoma and 1 case of acute leukemia).

We classified the patients into two age groups, prepubertal (0–11 years) and postpubertal (12–18 years), based on the typical onset of puberty in males around 12 years of age. The tumors had been assessed based on the criteria established by the WHO in 2022. The clinical stage was established according to the macroscopic aspect of primary tumors based on the guidelines of the WHO.

### 2.2. Immunohistochemical Analysis and Assessment Protocol

The immunohistochemical analysis used two monoclonal nuclear antibodies, anti-SALL4 (SALL4 Antibody, clone 6E3, source mouse, dilution 1:100, antigen retrieval- pH 9, BioSB) and anti-OCT3/4 (OCT3/4 Antibody, clone N1NK, source mouse, dilution 1:100, antigen retrieval- pH 6, Novocastra), after a 5-min pretreatment with proteinase K enzyme. We achieved immunoreaction detection using the UltraVision Quanto Detection System HRP DAB (ThermoFisher Scientific, Fremont, CA, USA). The evaluation of the markers was independently conducted by two pathologists. Nuclear expression of SALL4 and OCT3/4 proteins was considered positive because they bind to DNA and influence the transcription of target genes. Membranous and cytoplasmic staining was deemed negative because it does not reflect the functional localization of these proteins. We used a semi-quantitative four-tiered scoring system to evaluate the staining intensity: 0 for no staining, 1 for low-yellow, 2 for brown-yellow, and 3 for dark yellow. We evaluated the staining extent based on the percentage of positive tumor cells, assigning scores of 0 (less than 5%), 1 (5–25% positive cells), 2 (26–75% positive cells), and 3 (more than 76% positive cells) [11]. We calculated the final immunoreactivity score by summing the intensity and extent of the staining scores.

### 2.3. Statistical Analysis

Statistical data were collected using IBM SPSS Statistics 25, Microsoft Office Excel, and Word 2013. We tested quantitative variables using the Shapiro–Wilk test and expressed them using either means and standard deviations or medians with interpercentile ranges. Independent quantitative variables with normal distributions were compared between two independent groups using Student’s *t*-test and Welch’s *t*-test, while independent quantitative variables with non-parametric distributions were compared between groups using the Mann–Whitney U test. Qualitative variables were presented as absolute frequencies and percentages, and differences between groups were tested using Fisher’s exact test. We used Bonferroni-corrected Z-tests to detail the results obtained in contingency tables. We described overall survival (OS) and progression-free survival (PFS) in the overall cohort and between groups, reporting them as means with 95% confidence intervals (CI). Comparisons between groups for OS and PFS values were conducted using the Tarone–Ware test.

The primary outcomes of the study were focused on the identification of clinical and evolutive data of patients with a TT and the correlation of these data with the expression of SALL4 and OCT3/4 markers to understand the molecular mechanisms and develop effective diagnostic strategies. By achieving these objectives, the study proposed to standardize the expression of biomarkers in TT pathology, to confirm their role as prognostic or predictive factors, and improve the therapeutic management of patients.

## 3. Results

### 3.1. Patients’ Clinical and Pathological Characteristics

Our study included a total of 18 patients (Table 1). The most common symptoms were hemiscrotal enlargement (94.4%) and testicular pain (27.8%), which were more frequent in patients aged 12–18 years (62.5%) than in those aged 0–11 years (0%) (*p* = 0.007). Elevated alpha-fetoprotein (AFP) levels were observed in 61.1% of patients, and elevated human chorionic gonadotropin (hCG) in 33.3% of patients, which was significantly more frequent in patients aged 12–18 years compared to those aged 0–11 years (100% vs. 30%, 75% vs. 0%, *p* = 0.004/*p* = 0.002). The majority of patients underwent an orchiectomy (88.9%) and conservative surgery was initially performed for three patients in the prepubertal group, but two of these patients required a necessary radical orchiectomy due to unfavorable pathological results showing tumor cells. The patient diagnosed with cystic dysplasia underwent conservative surgery. Of the patients, 83.3% received chemotherapy (CHT) (80% with one protocol, 20% with two protocols; session average was 7.73 ± 4.25, median = 8). All patients with a GCT received cisplatin-based CHT. Most of the TTs were GCTs (83.3%), primarily mixed GCTs (seven cases, 39.7%), significantly more frequent in postpubertal patients compared to prepubertal patients (75% vs. 10%). Immature teratomas (IT) (four cases, 22.2%) were more frequent in patients aged 0–11 years compared to those aged 12–18 years (40% vs. 0%) (*p* = 0.005). A smaller percentage of GCTs were YSTs (three prepubertal cases, 16.7%) and there was only one case of embryonal carcinoma (EC) (5.6%). Even though paratesticular rhabdomyosarcoma (PT-RMS) is not a TT, we included these two patients in our study due to the paratesticular mass. Regarding staging, the majority of tumors were stage pT3 (33.3%), pT2 (27.8%), pT1a (22.2%), pT1b (11.1%), and pT4 (5.6%). The mean PFS was significantly shorter in patients aged 12–18 years compared to those aged 0–11 years (*p* = 0.047). The OS rate was 94.4%, while the PFS rate was 77.8%.

### 3.2. Patient’s Distribution Based on SALL4/OCT3/4 Immunostaining and Tumor Type and Subtypes

**Figure 1:**
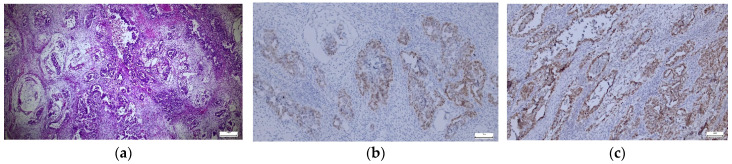
(**a**–**c**). Germinal tumor—hematoxylin–-eosin (HE) and OCT3/4, SALL4 immunostaining expression in tumor cells. (**a**) Germinal tumor—malignant structures with mature and immature nervous structures, cytonuclear atypia, atypical mitoses (×5). (**b**) OCT3/4—moderate positive nuclear expression in tumor cells (×10). (**c**) SALL4—diffuse positive nuclear expression in tumor cells (×10).

**Figure 2:**
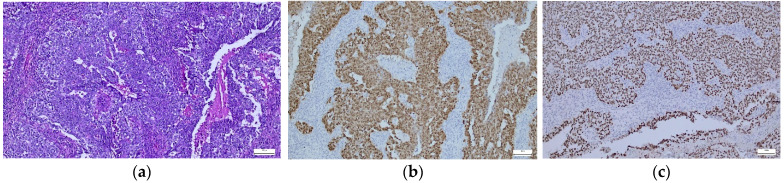
(**a**–**c**). Mixed germinal tumor—HE and OCT3/4/SALL4 immunostaining expression in tumor cells. (**a**) Mixed GCT with YST features, small areas with the appearance of a seminoma, necrosis, and hemorrhage (×5). (**b**) Tumor cells diffusely positive for OCT 3/4 (×10). (**c**) Cell tumor diffusely positive for SALL4 (×10).

The distribution of patients based on the investigated markers and age group indicates that 83.3% of tumors were positive for SALL4. OCT3/4 was positive in 38.9% of tumors, with a significantly higher prevalence in patients aged 12–18 years compared to those aged 0–11 years (75% vs. 10%, ***p* = 0.013**). The differences observed between IHC-positive staining and tumoral type were not statistically significant according to Fisher’s tests (Table 2). However, there was a trend toward statistical significance for the association of GCTs (Figure 1a–c) with positive SALL4 (*p* = 0.056). Based on positive staining and tumor subtype, the results indicated that the frequency of IHC positivity was significantly different only for OCT3/4 (***p* = 0.003**). Bonferroni-corrected Z-tests showed that patients with mixed GCTs (Figure 2a–c) were significantly more frequently associated with OCT3/4 positivity (85.7% vs. 9.1%). The frequency of marker immunostaining was not significantly different in relation to tumor staging for any of the analyzed markers, according to Fisher’s tests (*p* > 0.05). After analyzing the final IHC scores of the markers that were used to classify the tumors, the final sore for SALL4 was 5.33 ± 1.04 (mean ± SD), and the OCT3/4 final score was 5.14 ± 1.21 (mean ± SD), both markers having a median (IQR) of 6 (4–6). A high immunostaining expression for SALL4 was observed primarily in YSTs (score of 6 in all cases) and EC (score of 6), followed by mixed GCTs (mean ± SD = 5.57 ± 1.13), and PT-RMS (score of 5), with lower expression seen in IT (score of 4). OCT3/4 immunostaining was positive exclusively in GCT, with high expression in EC (score of 6) and mixed GCTs (mean ± SD = 5 ± 1.26).

### 3.3. Patient’s Distributions Based on SALL4/OCT3/4 Immunostaining versus Clinical and Pathological Characteristics

Positive IHC staining was not significantly associated with elevated AFP and LDH levels for any of the analyzed markers, according to Fisher’s tests (*p* > 0.05). Based on the investigated markers and hCG levels, statistically significant differences between groups were observed only for OCT3/4 values according to Fisher’s tests (***p* = 0.013**), with patients having high hCG levels being significantly more frequently associated with positive OCT3/4 values (71.4% vs. 9.1%).

The frequency of positive IHC markers was not significantly associated with the number of CHT regimens for any analyzed marker, according to Fisher’s tests (*p* > 0.05). Regarding the comparison of the number of CHT sessions relative to the investigated markers, statistically significant differences between groups were observed only for OCT3/4 values according to the Student’s test (***p* = 0.016**), with patients who were OCT3/4 positive having a significantly lower number of CHT sessions (4.67 ± 2.94) compared to patients who were OCT3/4 negative (9.78 ± 3.8).

According to Fisher’s exact tests, the differences between groups of patients based on the marker immunostaining and their outcome, recurrence, and death were not statistically significant for any of the markers (*p* > 0.05). As a result, there were no significant associations between the presence of subsequent metastases and the markers analyzed in the study.

## 4. Discussion

There is a scarcity of worldwide research on molecular markers in pediatric testicular cancers, making it crucial to understand their potential in this rare but clinically important pediatric malignancy. In our analysis, we performed immunostaining for SALL4 and OCT3/4 in 18 pediatric TTs and underlined the importance of these specific molecular markers as a diagnostic tool, especially those that present with a morphologic overlap and provide a definitive diagnosis.

OCT3/4 is a sensitive and specific marker for identifying intratubular germ cell neoplasia (IGCNU) in challenging cases and at-risk patients [12,13]. A total of 6 of the 157 patients who underwent testicular biopsies for reasons other than suspected testicular malignancies showed OCT4 nuclear staining in their germ cells. In these cases, three of the six patients were children with cryptorchidism, making the significance of OCT4 positivity less certain. The low incidence of positive OCT3/4 markers in adult infertility suggests that OCT3/4 immunostaining should not be routinely used in testicular biopsies for infertility unless there are additional risk factors present [14,15]. In our cohort, the presence of an intratesticular tumor warranted a comprehensive diagnostic approach. Using IHC staining with SALL4 and OCT3/4, we ensured a thorough evaluation, and the negative results for these markers supported the benign nature of testicular cystic dysplasia.

In accordance with a previous study, a more extensive analysis by Linde et al. evaluated the ability of OCT3/4 and D2-40 to detect ITGCN in boys older than 2 years with cryptorchidism. Sections from 309 testicular biopsies from 234 boys aged 1 month to 14 years, surgically treated for cryptorchidism, were tested with PLAP, OCT3/4, CD117, and D2-40. The prevalences of positive OCT3/4 markers in germ cells in testicular biopsies were different for each age group and commonly seen below 12 months old, where most orhidopexies are performed. None of the 192 testes, except one from boys older than 2 years, had OCT3/4 or D2-40 positive germ cells identified [16]. In our study, OCT3/4 was positive in 38.9% of tumors, with a significantly higher prevalence in postpubertal patients than prepubertals. Our study and the Linde et al. study both highlight the context-specific utility of OCT3/4 staining. While Linde et al. demonstrate the marker’s limited value in older children with cryptorchidism, our study underscores its importance in diagnosing malignant TTs, particularly in older pediatric patients. This comparison reinforces the need for targeted application of OCT3/4 based on patient age and clinical context, ensuring accurate diagnosis and effective clinical management.

Only a handful of studies have demonstrated the usefulness of OCT3/4 IHC in the diagnostic setting of TTs or testicular biopsies. In a large series of 209 GCTTs investigated after radical orchidectomy, all seminomas and EC were all consistently positive for OCT3/4. None of the three spermatocytic seminomas or three non-GCTs (embryonal RMS) were stained for OCT3/4 [17]. This aligns with our results and underscores the significant diagnostic value of OCT3/4 in identifying mixed GCTs with components of seminoma or EC cells. The high expression of OCT3/4 as a sensitive marker in EC or EC cells as a component in most mixed GCTs could explain the general high CHT sensitivity and curability of these malignancies. Mueller et al. conducted research suggesting that a loss of OCT3/4 expression induces a higher apoptotic threshold and cisplatin resistance in EC cells of nonseminomatous TGCTs [18]. In our series, we had only one case of mixed GCT with components of EC and YSTs with a loss of OCT3/4 expression and subsequent cisplatin resistance, resulting in death 17 months after diagnosis, which aligns with Mueller et al.‘s results. Also, patients who were OCT3/4-positive received a significantly lower number of CHT sessions compared to patients who were OCT3/4-negative. This highlights the importance of OCT3/4 as a molecular marker in both diagnostic and therapeutic contexts, emphasizing its potential role in guiding personalized treatment strategies to improve patient outcomes.

OCT3/4-positive staining was seen in testicular intraepithelial neoplasia (TIN) loci in 75% of paratumorus tissue, while the detection rate of TIN with only HE staining was 34% [19]. This result underscores the urgent need to use IHC in TT. Iczkowski et al. supported our findings that in the case of EC, OCT3/4 exhibited greater specificity; they compared it to CD30 and found it to be superior to PLAP [10,20]. Only a few studies supported the extension of OCT3/4 testing to pediatric cases and identified this marker as a useful tool in the diagnosis of a malignant GCT [21]. Our research aligns with results from a study on 47 children from a total of 145 GCTTs, which demonstrated that OCT3/4 may be a better predictive marker to distinguish TGCT. OCT3/4 was positive in 70.3% (102/145) of all TGTCs, including 27% (9/33) in mature teratoma and 100% (14/14) in mixed GCTs. In EC and seminoma, OCT3/4 was 100% positive and negative in all IT and YST [22].

Designing a novel, concise, and affordable IHC panel is necessary to differentiate GCTs. Some research conducted a series of panels constituting OCT 3/4, CD117, GPC3, and CD30 with good sensitivity and specificity in differentiating seminomas, YSTs, and EC, respectively [23]. This result highlights the potential for a concise and affordable panel to aid in the differential diagnosis of TTs, providing clinicians with valuable information for treatment planning and patient management. In the future, further research and validation of such panels could improve their clinical utility and impact on patient outcomes.

In a large series of 3215 tumors, SALL4 was consistently expressed in all GCTs except some trophoblastic tumors and mature components of teratomas. SALL4 immunostaining was 100% in testicular seminoma, EC and YSTs, 84% in choriocarcinoma, and 60% in mature teratoma. An expression of SALL4 was occasionally detected in mesenchymal and neuroectodermal neoplasm, and only in one case of embryonal rhabdomyosarcoma (1/43, 2%) [6]. In our cohort, we observed SALL4 positivity in 83.3% of tumors, showing a trend towards statistical significance with GCT association. We observed unexpected findings of SALL4 positivity in a patient with PT-RMS, with an unfavorable outcome characterized by the development of subsequent metastases necessitating a second regimen of CHT. SALL4 positivity in RMS is not typical and is an uncommon occurrence. This case underscores the complexity of tumor biology and the need for comprehensive diagnostic approaches in rare and atypical presentations of pediatric TTs. Incorporating markers like SALL4 into diagnostic panels may aid in refining treatment strategies and improving outcomes for patients with these challenging malignancies.

In a study of thirteen pediatric GCTTs, AFP and SALL4 were expressed in all seven YSTs. The majority of teratomas expressed SOX2 and PDPN, whereas SALL4 was found in 8/13 immature teratomas. They analyzed a pattern of multiple IHC markers in all types of GCTs (AFP, hCG, PLAP, SALL4, OCT3/4, AP-2 γ, GATA4, SOX2, PDPN, MAGE-A4, and AMH), and their results indicated that the expression pattern of these antigens is similar between pediatric and adult GCTs. Regarding differences in gene expression between pediatric and adult GCTs, this remains an unanswered question and a key to achieving the goals of precision medicine [24]. Our analysis revealed a high SALL4 IHC expression with a maximum score in YSTs and EC, followed by mixed GCTs and PT-RMS, with lower expression in ITs.

There were several functional associations between SALL4 and cancer incidence, progression, and subsequent metastasis, suggesting that this molecular marker could be effective in eradicating malignant cells [25]. Several studies have established that SALL4 overexpression stimulates proliferation, development, invasion, and migration in cancers, highlighting its critical oncogenic role in gene transcription and tumor growth. Understanding and studying the mechanisms of SALL4 in cancer may help to develop new therapeutic perspectives for rare cancer conditions [26]. Modern technologies like nanomedicine, which use nanoparticle carriers to transmit drums and siRNAs, have gained more attention in cancer treatment because of their high efficacy, safety, and ability to target specific tumors. Additional research in this area could potentially provide novel ideas and perspectives for SALL4-targeting cancer treatment [27].

As a retrospective study, our research encountered several limitations. These included insufficient and incomplete data regarding medical history and clinical examination, as well as the significant constraint of a small patient cohort. Limitations were exacerbated by the COVID-19 pandemic, which restricted patients’ access to regular evaluations. Long-term follow up was not achievable, as many patients reached 18 years of age and were lost to our observation, being redirected to Adult Oncology Centers. Additionally, the subjective nature of manual scoring methods used for marker expression assessment presents a potential limitation.

## 5. Conclusions

Our study underscores the diagnostic and prognostic utility of SALL4 and OCT3/4 immunostaining in pediatric TTs. The differential expression patterns of SALL4 and OCT3/4 highlight their potential as biomarkers to guide treatment decisions and predict outcomes. SALL4 exhibited a positive expression in the majority of TTs (83.3%), with the highest frequency in GCTs (93.3%). OCT3/4 showed positive expression in approximately 38.9% of TTs, with significantly higher prevalence in mixed GCTs (85.7%) compared to other tumor types (9.1%). Positive OCT3/4 expression was associated with a reduced requirement for chemotherapy sessions, compared with negative OCT3/4 expression which necessitated more sessions. Loss of OCT3/4 expression in our cohort correlated with a negative prognosis and resistance to cisplatin-based chemotherapy. This study underscores the importance of OCT3/4 as a predictive factor in pediatric TTs, highlighting its substantial correlation with tumor type and its impact on treatment response. Further research in pediatric TTs is warranted to validate these findings and explore the molecular mechanisms for better personalized therapeutic strategies.

## Figures and Tables

**Table 1:** Patients’ distributions according to clinical and pathological characteristics, reported by age.

Parameter/Age	Total	0–11 Years	12–18 Years	*p*
***Number of patients***	18 (100%)	10 (55.6%)	8 (44.4%)	-
***WHO Classification (No., %)***				
Germinal Cell Tumor	15 (83.3%)	8 (80%)	7 (87.5%)	1.000 *
PT-RMS	2 (11.1%)	1 (10%)	1 (12.5%)	
Testicular cystic dysplasia	1 (5.6%)	1 (10%)	0 (0%)	
***Serum tumor markers (STM) (No., %)***				
Elevated AFP	11 (61.1%)	3 (30%)	8 (100%)	0.004 *
Elevated hCG	6 (33.3%)	0 (0%)	6 (75%)	0.002 *
Elevated LDH	7 (38.9%)	5 (50%)	2 (25%)	0.367 *
***Chemotherapy (No., %)***	15 (83.3%)	8 (80%)	7 (87.5%)	1.000 *
***Outcome (No., %)***				
Unfavorable	4 (22.2%)	1 (10%)	3 (37.5%)	0.275 *
Favorable	14 (77.8%)	9 (90%)	5 (62.5%)	
***Metastasis at admission (No., %)***	2 (11.1%)	0 (0%)	2 (25%)	0.183 *
***Subsequent metastasis (No., %)***	4 (22.2%)	1 (10%)	3 (37.5%)	0.275 *
***Recurrence (No., %)***	2 (11.1%)	1 (10%)	1 (12.5%)	1.000 *
***Death (No., %)***	1 (5.6%)	0 (0%)	1 (12.5%)	0.444 *
***Follow-up period***				
Mean ± SD	70.06 ± 71.35	114.4 ± 67.55	14.63 ± 13.34	0.001 ***
Median (IQR)	33.5 (8.7–124)	113 (61–179.5)	13 (2.75–20.7)	
**OS (months) (Mean (95% C.I.))**	194.23 (166.41–222)	209	35 (24.8–45.1)	0.134 **
**PFS (months) (Mean (95% C.I.))**	155.18 (109.6–200.7)	187.33 (147.3–227.3)	14 (8.15–19.84)	0.047 **

* Fisher’s Exact Test, ** Tarone–Ware Test, *** Welch *t*-test. PT-RMS: paratesticular rhabdomyosarcma; AFP: alpha-fetoprotein; hCG: human Chorionic Gonadotropin; LDH: lactate dehydrogenase; OS: overall survival; PFS: progression free survival.

**Table 2:** Patient’s distributions based on SALL4/OCT3/4 and histological subtypes.

*Tumor Subtype* *SALL4*	*SALL4 Negative*		*SALL4 Positive*		*p* *
	Nr.	%	Nr.	%	
Cystic Dysplasia	1	33.3%	0	0%	0.109
EC	0	0%	1	6.7%	
IT	1	33.3%	3	20%	
Mixed GCT	0	0%	7	46.7%	
PT-RMS	1	33.3%	1	6.7%	
YST	0	0%	3	20%	
***Tumor subtype*** ***OCT3/4***	***OCT ¾ negative***		***OCT ¾ positive***		*p* *
	Nr.	%	Nr.	%	
Cystic Dysplasia	1	9.1%	0	0%	**0.003**
EC	0	0%	1	14.3%	
IT	4	36.4%	0	0%	
Mixed GCT	**1**	**9.1%**	**6**	**85.7%**	
PT-RMS	2	18.2%	0	0%	
YST	3	27.3%	0	0%	

* Fisher’s Exact Test. EC: embryonal carcinoma; IT: immature teratoma; GCT: germ cell tumor; PT-RMS: paratesticular rhabdomyosarcoma; YST: Yolk sac tumor.

## Data Availability

Data are contained within the article.
